# The Exocyst Complex in Health and Disease

**DOI:** 10.3389/fcell.2016.00024

**Published:** 2016-04-12

**Authors:** Magdalena Martin-Urdiroz, Michael J. Deeks, Connor G. Horton, Helen R. Dawe, Isabelle Jourdain

**Affiliations:** Biosciences, College of Life and Environmental Sciences, University of ExeterExeter, UK

**Keywords:** exocyst complex, fungi, plants, mammals, pathogens

## Abstract

Exocytosis involves the fusion of intracellular secretory vesicles with the plasma membrane, thereby delivering integral membrane proteins to the cell surface and releasing material into the extracellular space. Importantly, exocytosis also provides a source of lipid moieties for membrane extension. The tethering of the secretory vesicle before docking and fusion with the plasma membrane is mediated by the exocyst complex, an evolutionary conserved octameric complex of proteins. Recent findings indicate that the exocyst complex also takes part in other intra-cellular processes besides secretion. These various functions seem to converge toward defining a direction of membrane growth in a range of systems from fungi to plants and from neurons to cilia. In this review we summarize the current knowledge of exocyst function in cell polarity, signaling and cell-cell communication and discuss implications for plant and animal health and disease.

## Introduction

Exocytosis involves the fusion of intracellular secretory vesicles with the plasma membrane (PM), thereby delivering integral membrane proteins at the cell surface and releasing material into the extracellular space, such as hormones or components of the extracellular matrix. Importantly, exocytosis also provides a source of lipid moieties for membrane extension. The addition of new membranes to specific areas of the cell periphery is a fundamental requirement for growth, polarity, or division and is therefore critical for cell function and tissue development.

A further understanding of the mechanisms regulating secretory vesicle sorting, transport, and targeting is beginning to deepen. Cargoes emanating from intracellular endomembrane compartments are transported by motor proteins along cytoskeletal tracks toward polarized areas of the PM. The initial contact between the vesicle and the PM (aka tethering) is mediated by the exocyst complex, which consists of eight proteins named Sec (for “secretion”) or Exo (for “exocyst related”): Sec3, Sec5, Sec6, Sec8, Sec10, Sec15, Exo70, and Exo84 (TerBush and Novick, 1995; TerBush et al., 1996). The exocyst complex, aided by Sec1/Munc-18, is thought to bridge the SNAREs (soluble N-ethylmaleimide-sensitive factor attachment protein receptor) on opposing membranes (2 target t-SNAREs and 1 vesicular v-SNARE) (Hong and Lev, 2014). This paired trans-SNARE complex docks the vesicle to the receiving membrane and finally induces lipid fusion.

The exocyst complex has been extensively studied using *Saccharomyces cerevisiae* as a model organism. But even within the budding yeast community, the mechanisms of its assembly, and mode of action are controversial. It was originally proposed that the assembly of the exocyst complex is sequential. In this model, Sec3 and Exo70 sit at the PM and this localization is independent of the secretion and transport machineries (Finger et al., 1998). At the PM, Sec3, and Exo70 interact with phosphatidylinositol (4,5)-bisphosphate (PIP2) (Boyd et al., 2004; He et al., 2007; Liu et al., 2007; Pleskot et al., 2015) and assemble the rest of the exocyst complex, which is trafficked to the PM on vesicles in an actin-dependent manner (Jin et al., 2011). For these reasons, Sec3 and Exo70 were described as landmarks for polarized secretion (Boyd et al., 2004). This model implies that at least two sub-complexes independently exist prior to the formation of the full complex, one at the PM and one on the secretory vesicle. However, convincing evidence later indicated that the endogenous untagged Sec3 protein behaves similarly to the other exocyst subunits, thus calling the landmark model into question (Roumanie et al., 2005). Furthermore, the fully assembled octameric complex, but not any sub-complex, could be isolated from *S. cerevisiae*, in the most advanced biochemical study of the exocyst complex to date (Heider et al., 2016).

The complex is highly conserved across evolution (Koumandou et al., 2007) and it was long assumed that the properties and mechanisms of action of the exocyst in budding yeast also applied to other organisms. In recent years, the interest that the exocyst has raised in fungi, plants and animal systems, has unraveled new functions, similarities and differences with the paradigmatic yeast model. This review aims to summarize some of them, and to further highlight the importance of the exocyst complex in normal cell function and pathological situations. The interplay between the exocyst and its regulators have been extensively reviewed elsewhere (e.g., Wu et al., 2008; Guichard et al., 2014; Mukherjee et al., 2014; Shirakawa and Horiuchi, 2015) and are not emphasized here.

## The fungal exocyst complex

### The exocyst complex in fungi

Fungi have a single homolog encoding gene for each of the eight exocyst components (TerBush and Novick, 1995; TerBush et al., 1996; Guo et al., 1999, 2015; Wang et al., 2002, 2003; Li et al., 2007; Kohli et al., 2008; Taheri-Talesh et al., 2008; Panepinto et al., 2009; Jones and Sudbery, 2010; Bendezu et al., 2012; Jourdain et al., 2012; Giraldo et al., 2013; Irieda et al., 2014; Kwon et al., 2014; Riquelme et al., 2014; Chavez-Dozal et al., 2015a,b; Gupta et al., 2015), and most of these genes are essential for viability except *exo70* in *Schizosaccharomyces pombe, sec3* in *S. cerevisiae, Candida albicans*, and *Aspergillus niger, sec5* in *Neurospora crassa*, and both *exo70* and *sec5* in *Magnaporthe oryzae* (Finger and Novick, 1997; Wang et al., 2002; Li et al., 2007; Kohli et al., 2008; Kim D. U. et al., 2010; Giraldo et al., 2013; Kwon et al., 2014; Riquelme et al., 2014). Thus, the same component of the exocyst may not share identical interactions within the complex in different fungi. However, co-purification and co-immunoprecipitation experiments conducted in *N. crassa* and in *M. oryzae* indicate that in these two fungi at least, the exocyst exists as an octameric complex (Riquelme et al., 2014; Gupta et al., 2015). All members of the exocyst have a similar fold made of long helical bundles, which are thought to pack against one another to form the exocyst complex (Munson and Novick, 2006; Heider et al., 2016). It is therefore possible that structural redundancies exist between fungal species.

### The exocyst in normal polarized hyphal growth

**Figure 1:**
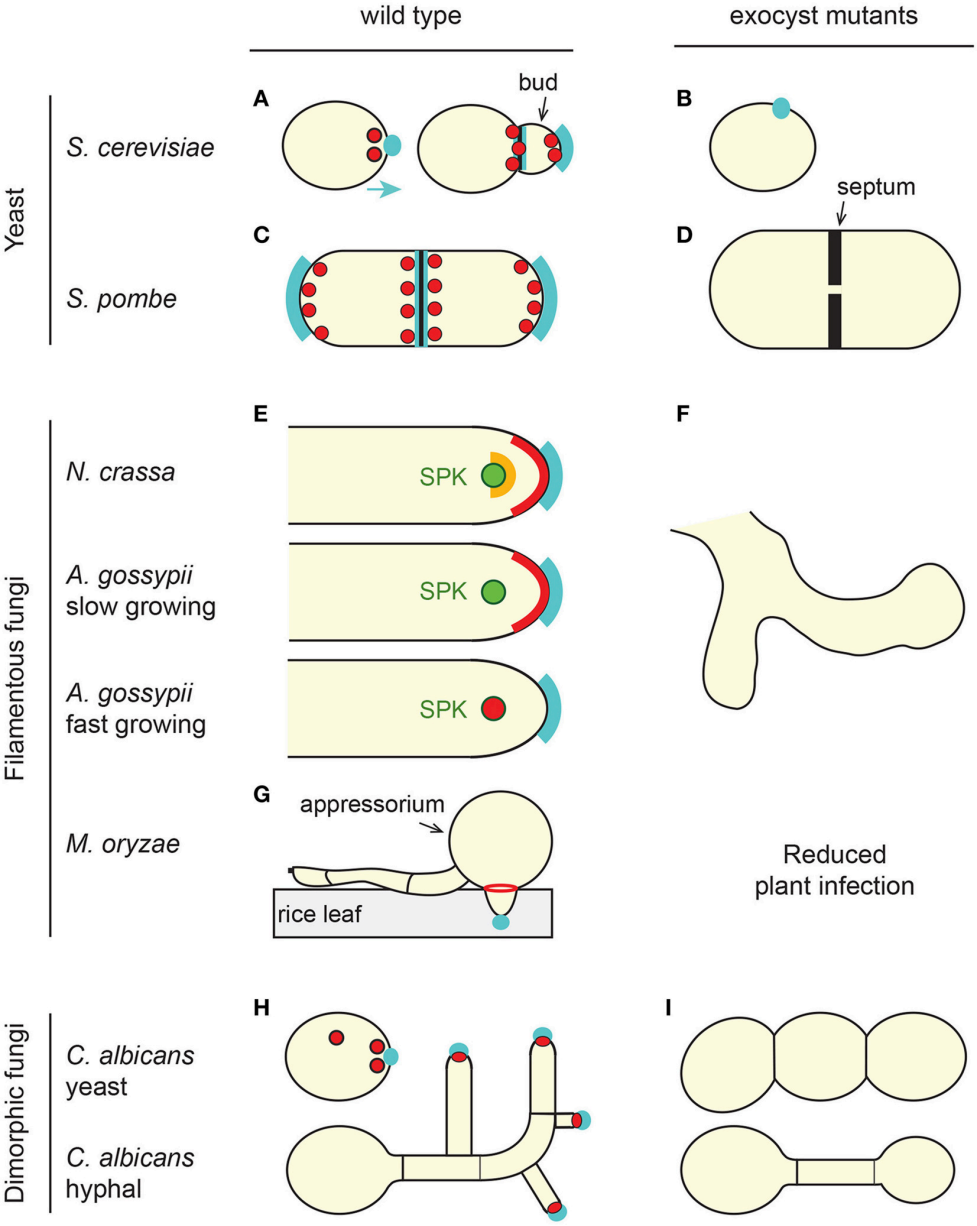
**The exocyst complex in fungi. (A,C,E,G,H)** Overall localization of the exocyst complex (red and orange) in wild-type cells of representative fungi. For simplicity, *S. pombe* cells **(C,D)** are represented as both elongating and dividing. In all fungi, the exocyst localizes at sites of polarized secretion and polarized growth (cyan), the bud tip in budding cells **(A,H)**, cell tip area in tip-growing fungi **(C,E)** or septal area in dividing cells **(A,C)**. In some cases individual sub-units or sub-complexes are present at different sub-cellular locations, which is only illustrated here for *N. crassa* (**E**, red and orange). The exocyst localizes as a ring at the base of the *M. oryzae* appressorium during rice leaf infection **(G)**. SPK, Spitzenkörper (green). **(B,D,F,I)** Typical cellular phenotype of exocyst mutants. In the absence of a functional exocyst, most fungal species have a morphological or loss of polarity phenotype, failing to grow a bud **(B)**, growing wider **(D)**, branching out **(F)**, or failing to branch **(I)**. Note that in the case of the hyphal form of *C. albicans*, three different phenotypes were described. We arbitrarily present here a hypo-branching and globular tip phenotype reported for a *sec3* defective mutant. Some fungi also have a cytokinetic defect **(D,I)**. See text for details and references.

Fungi exhibit different modes of growth with a wide variety of sizes and shapes (Figure 1). Filamentous fungi such as *N. crassa, Ashbya gossypii, Aspergillus nidulans, Fusarium oxysporum*, or *M. oryzae* form hyphae. Hyphae are long cylindrical structures that show sustained polar growth at their tips and that are able to form a branched network of hyphae (aka mycelium). In multicellular hyphae, individual cells are separated by a septum, but aseptated, unicellular hyphae also exist. Yeast are unicellular fungi that do not normally form hyphae. The fission yeast *Schizosaccharomyces* (e.g., *S. pombe*) are rod- shaped, and like filamentous fungi, grow by tip extension and form a septum perpendicular to the cell's long axis. By contrast, budding yeast (e.g., *S. cerevisiae)* are round or ovoid and divide by budding. Dimorphic fungi (e.g., *C. albicans, Ustilago maydis*, or *Zymoseptoria tritici)* are capable of switching from a yeast form to a hyphal or pseudohyphal form, where the pseudohyphae are branching chains of cells that fail to separate after mitosis. In yeast and most common model fungi, non-essential genes can be fully deleted and for genes that are essential for viability, conditional mutants can be generated (the fungus is only mutant under certain experimental conditions). Thus, a number of mutants of the exocyst complex have been created in various fungal species (TerBush and Novick, 1995; TerBush et al., 1996; Guo et al., 1999; Wang et al., 2002, 2003; Li et al., 2007; Bendezu et al., 2012; Jourdain et al., 2012; Giraldo et al., 2013; Kwon et al., 2014; Riquelme et al., 2014; Chavez-Dozal et al., 2015a,b; Gupta et al., 2015). This has allowed the identification of the different cellular functions of the exocyst complex in fungi.

The filamentous growth is an extreme polarized process that requires the selection of one growth site and its maintenance by selective transport and targeting of secretory vesicles toward what will constitute the hyphal tip. At the apex, these secretory vesicles are incorporated to specific domains of the PM, where they allow its expansion. They ensure the delivery of material and enzymes involved in the synthesis of the fungal cell walls and the release of proteins to the extracellular space. The polarization site is marked by polarity cues that enable the assembly of the machinery responsible for polar growth. Once the site has been chosen, the multiprotein complex termed the polarisome is deposited at cell tips by microtubules (MTs) (Feierbach et al., 2004; Takeshita et al., 2008; Takeshita and Fischer, 2011) and orchestrates the nucleation of actin cables by formins, along which the secretory vesicles are transported (Pruyne and Bretscher, 2000a,b; Pruyne et al., 2002). At the hyphal tip, fungi have a Spitzenkörper (SPK, “tip body”), a sub-apical membrane rich region where secretory vesicles continuously accumulate prior to fusion with the PM (Riquelme et al., 2007; Verdin et al., 2009; Sanchez-Leon et al., 2011; Richthammer et al., 2012; Sanchez-Leon and Riquelme, 2015). The SPK is also composed of ribosomes, MTs, actin microfilaments, ER and Golgi equivalents (GEs) and an amorphous material of undefined nature (Girbardt, 1969; Grove and Bracker, 1970; Howard and Aist, 1979; Howard, 1981; Bourett and Howard, 1991; Roberson and Vargas, 1994; Steinberg, 2007; Pinar et al., 2013). Although it is not an organelle in strict sense, the SPK is a very dynamic apical cluster that behaves as a single unit to drive direction and rate of growth at the hyphal apex (Bartnicki-García, 2002; Araujo-Palomares et al., 2007; Kohli et al., 2008; Taheri-Talesh et al., 2008) and can be seen as a Vesicle Supply Center (VSC) (Bartnicki-Garcia et al., 1989, 2000; Bartnicki-García, 1990). After their accumulation in the SPK and before fusion with the PM, the SPK vesicles interact finally with components of the polarized growth machinery, such as the exocyst complex (Kohli et al., 2008; Riquelme et al., 2014).

It is commonly accepted that exocyst components are at the apex of growing fungal hyphae or yeast ends, forming a crescent that follows the curvature of the tip (Sudbery, 2011). Systematic and comparative observations suggest that the spatio-temporal localization of the exocyst complex subunits is probably more complex, and possibly fungus-dependent (Figure 1). In hyphae of *C. albicans, A. nidulans, A. oryzae, M. oryzae*, and *Z. tritici*, all the exocyst components studied localize to their hyphal tip surface but this localization is not associated to the position of the SPK (Taheri-Talesh et al., 2008; Jones and Sudbery, 2010; Hayakawa et al., 2011; Sudbery, 2011; Giraldo et al., 2013; Guo et al., 2015; Gupta et al., 2015). In *A. gossypii*, the sub-cellular localization of Exo70, Sec3, and Sec5 depends on the rate of hyphal growth. In slow-growing hyphae, they form a cap at the tip of the hyphae while in fast growing hyphae they accumulate at the SPK (Kohli et al., 2008). In interphase fission yeast cells, all subunits co-localize at cell poles but in the form of patches rather than an apical crescent (Wang et al., 2002; Bendezu et al., 2012; Jourdain et al., 2012). They are also seen on cytoplasmic trafficking vesicles (Bendezu et al., 2012). By contrast, and regardless of the growth rate, in mature hyphae of *N. crassa* four subunits localize in a crescent at the foremost apical region of the hyphae, two subunits accumulate at the SPK outer layer and SEC-3 is at the interface between these two structures (Riquelme et al., 2014). Moreover, the localization of SEC-6 at the PM, but not EXO-70, is independent of the secretory pathway. This is in marked contrast to the situation in *S. cerevisiae*, where SEC3 and EXO70 are associated with the PM and do not depend on the secretory pathway, whilst other subunits are carried on the secretory vesicles (Boyd et al., 2004). These findings support the hypothesis that sub-complexes exist in some, but possibly not all fungi. The results further indicate that different members of the exocyst may act as landmarks for polarized secretion in different fungi and that the mode of assembly of the exocyst complex may differ between fungi.

In yeast where travel distances are short (approx. 10 μm), the vesicular subunits of the exocyst complex are trafficked toward the cell periphery by an actin-based mechanism (Boyd et al., 2004; Jones and Sudbery, 2010; Bendezu et al., 2012). In filamentous fungi distances are much longer (approx. 100 μm in hyphae from *U. maydis*, Schuster et al., 2011) and MTs organize the long-range transport of exocyst-carrying vesicles whereas actin distributes vesicles locally at the cell tip (Kohli et al., 2008; Hayakawa et al., 2011; Riquelme et al., 2014). The SPK may constitute the transition zone where vesicles switch from MTs to actin (Harris et al., 2005; Riquelme et al., 2014). This is in a manner reminiscent of the situation in neurons where actin-based motors support the switch of endomembrane compartments from MTs to the actin-rich periphery (Woolner and Bement, 2009). Hence, the mode of transport of the exocyst subunits in filamentous fungi may simply depend on the travel distance.

The interdependence of the polarisome, the SPK and the exocyst to orchestrate hyphal tip expansion is evidenced by their relative localization at the hyphal tips. However, this relative localization exhibits differences within fungi and suggests variable modes of interplay between the three components. In *N. crassa*, EXO-70, and EXO-84 partially co-localize with the outer layer of the SPK (Verdin et al., 2009) and suggest that the exocyst is involved in the delivery of the vesicles from the SPK to the PM (Riquelme et al., 2014). In this organism, the polarisome component SPA-2 co-localizes and interacts with the SPK and spreads radially from there to the PM (Araujo-Palomares et al., 2009). A change in the position of the SPK displaces SPA-2 (Araujo-Palomares et al., 2009). In *M. oryzae*, the SPK marker Mlc1 co-localizes with FM4-64 in what it constitutes the SPK (Giraldo et al., 2013). In this fungus, Spa2 forms a bright spot at the hyphal tips in a structure similar to that formed by the SPK, while all the exocyst components are forming a cap at the PM (Gupta et al., 2015). In *C. albicans*, the normal localization of the SPK, the polarisome and the exocyst is disturbed by the lack of Sec15 however, the three components re-localized after *Sec15* is expressed again (Jones and Sudbery, 2010; Chavez-Dozal et al., 2015b). By contrast, mutations in fission yeast Sec3 affect the localization of the formin For3 but the rest of the polarisome remains unaffected (Jourdain et al., 2012).

In fungal mutants of the exocyst, post-Golgi vesicles accumulate in the cytoplasm and secretion is affected (e.g., Panepinto et al., 2009; Chavez-Dozal et al., 2015a). As a result, cell wall components are abnormally deposited and confer sensitivity to cell wall stressors or degrading enzymes (Panepinto et al., 2009; Chavez-Dozal et al., 2015a,b). Furthermore, Caballero-Lima and co-workers proposed a model for *C. albicans* hyphal tip growth in which the density of exocyst components determine the rate of cell wall-synthesizing-containing vesicles that fuse with the hyphal tip (Caballero-Lima et al., 2013). In yeast, like in other higher eukaryotes, the exocyst is critical for cytokinesis and cells usually die because of a failure to build two new PM and cell walls (aka septum) between the two daughter cells (Wang et al., 2002, 2003; Fendrych et al., 2010; Neto et al., 2013b; Chavez-Dozal et al., 2015a; Perez et al., 2015). In fission yeast, the endo-β-glucanases required for cell separation during cytokinesis are located at the septum in a process mediated by the exocyst (Martin-Cuadrado et al., 2005). Accordingly, cells in which the exocyst complex is mutated fail to divide and often develop multiple thick septa (Wang et al., 2002; Jourdain et al., 2012) (Figure 1D). These cells are also wider than the wild-type and show an accumulation of secretory vesicles that do not fuse with the PM at cell tips (Jourdain et al., 2012). Surprisingly however, they do not seem to have any other cell wall defects besides a thickening of their septa (Jourdain et al., 2012). More specifically, it has been reported that the localization of the exocyst components in the cortex of *S. pombe*, and not cell shape or cell wall synthesis regulators, defines its growth pattern (Abenza et al., 2015). Similarly equatorial, but not tip endocytosis is affected in a mutant of *sec8* (Gachet and Hyams, 2005). Thus, in fission yeast, the exocyst complex may play a different role during polarized cell growth and during cytokinesis. Mutant cells often show mating defects because they fail to secrete mating hormones, form a mating projection and develop a forespore membrane and cell wall (Wiederkehr et al., 2003; Sharifmoghadam et al., 2010). Fungal exocyst mutants are also characterized by the development of multiple new tips, which result in hyperbranching proposed to be due to the role that the exocyst plays in the organization of the SPK (Riquelme et al., 2014; Chavez-Dozal et al., 2015b) (Figure 1F). However, the involvement of the exocyst complex in cellular branching is likely to be more complex. Firstly, *S. pombe* cells can be “T-shaped” but the growth of a third pole does not depend on the exocyst. Secondly, in some cases loss of exocyst function in *C. albicans*—which normally has a SPK, and in neurons—which do not have a SPK, equally reduces cellular branching (Vega and Hsu, 2001; Chavez-Dozal et al., 2015a) (Figures 1I, **3B**).

In contrast to exocytosis, endocytosis is responsible for the uptake of extracellular material and the recycling of lipids and surface proteins. A large number of conserved endocytic proteins participate in the steps of endocytosis and their ordered recruitment and function have been extensively characterized (Kaksonen et al., 2006). Nevertheless, what defines the sites of endocytosis remains unknown. The idea of a spatiotemporal coordination of exocytosis and endocytosis is intuitive as cell shape, size or symmetry can only be achieved if membrane addition is balanced by subtraction. At the hyphal tip of *A. nidulans*, exocytosis (at the front) and endocytosis (at the rim) have been proposed to be spatially coupled, while in *C. albicans* a new model for hyphal growth has been developed in which the form of the hyphae can be predicted if cell wall synthase enzymes are removed or inactivated from subapical regions of the hyphal membrane (Taheri-Talesh et al., 2008; Caballero-Lima et al., 2013). In yeast, endocytosis happens in actin patches which are normally localized at sites of active growth or division (e.g., Gachet and Hyams, 2005). In exocyst mutants however, endocytic patches assemble but are delocalized and fail to internalize, and uptake of endocytic dyes is perturbed (Riezman, 1985; Gachet and Hyams, 2005; Jourdain et al., 2012; Jose et al., 2015). Moreover, members of the yeast exocyst complex genetically and physically interact with the endocytic machinery (Jourdain et al., 2012; Jose et al., 2015). This was also observed in higher eukaryotes (Sommer et al., 2005; Zuo et al., 2006). In fission yeast, the exocyst does not appear to co-localize with actin patches in an heterogeneous population of cells in which polarity is permanently maintained (Jourdain et al., 2012). The reason may be that endocytosis and exocytosis are coupled during polarity establishment, but not maintenance (Jose et al., 2015). In conclusion, the exocyst complex is ideally placed to coordinate the balance between endo- and exocytosis (Jourdain et al., 2012; Gupta et al., 2015; Jose et al., 2015). Directly or indirectly, the exocyst complex may in fact regulate all cellular actin structures- cables, patches and actomyosin rings. Besides a defect in actin patches distribution and function, the constriction and disassembly of the cytokinetic actomyosin ring is compromised in *S. pombe* mutants of *sec3* and these cells have no actin cables (Jourdain et al., 2012). In *S. pombe*, actin cables and the exocyst constitute two distinct but partially redundant morphogenetic pathways that contribute together to the robustness of the polarization machinery (Bendezu and Martin, 2011). The fact that Sec3 regulates both explains that *sec3* mutant cells are mis-shapened (Figure 1D). How these findings extend to filamentous fungi remains to be determined but the answer may lie in the organization of the actin-rich SPK (Riquelme et al., 2014; Chavez-Dozal et al., 2015b).

### The exocyst and fungal pathogenicity

Fungi are organisms with an enormous ecological and economic impact that in many ways are beneficial for humankind (Rokem, 2007). However, fungi can cause diseases in plants and animals having serious implications on human health and on the ecosystem (Fisher et al., 2012). The impact of fungal diseases is clearly manifested in crops. The rice blast fungus *M. oryzae* is one of the most destructive plant pathogens and in 2012 it was voted first of a Top 10 fungal plant pathogens list (Dean et al., 2012). This highlights the economic importance of this fungus that has become a model organism for the study of host-pathogen interactions.

*M. oryzae* infection of rice plants is mediated by an appressorium. This dome-shape specialized infection structure re-polarizes at its base where it generates the penetration hypha to rupture the leaf cuticle and invade the plant tissue. A report on the role of the exocyst complex during the appressorium-mediated infection by this plant pathogenic fungi has been recently published (Gupta et al., 2015). Through the generation of fluorescently tagged proteins, the different sub-units of the *M. oryzae* exocyst have been observed to assemble at the appressorium pore before emergence of the penetration peg (Figure 1G). This localization of the exocyst complex requires the prior formation of the septin ring (Dagdas et al., 2012) and indicates that polarized exocytosis is required for initiating polarity and protrusion of the penetration hypha. This result is consistent with the exocyst's role as a network hub, connecting endocytosis and exocytosis to optimize polarized growth (Jose et al., 2015). In fact, the targeted gene deletion of *exo70* and *sec5* results in a high reduction in the total protein secreted by the fungus, including the virulence-associated factor, spore tip mucilage (STM). In line with these results, Exo70 and Sec5 are required for full virulence of *M. oryzae* in plants and in a temperature-sensitive mutant of *sec6*, the disassembly of the exocyst complex causes a reduction in the virulence of the disease (Gupta et al., 2015).

During host invasion, pathogens secrete small molecules or effectors to suppress host immune responses and support pathogen growth. Based on their localization pattern, *M. oryzae* effector proteins can be classified in two groups. Apoplastic effectors accumulate extracellularly at the host-pathogen interface. By contrast, cytoplasmic effectors accumulate at a host derived structure termed the biotrophic interfacial complex (BIC) (Khang et al., 2010; Giraldo et al., 2013). In 2013, Giraldo et al. showed that apoplastic effectors are actively secreted via the conventional secretory pathway previously defined in filamentous fungi, and that cytoplasmic effectors are host-translocated by an unconventional mechanism involving the exocyst complex (Giraldo et al., 2013). The results obtained by Gupta et al. also suggest that disruption of the exocyst complex at the appressorium pore could inhibit secretion of virulence-associated proteins at later stages of appressorium development prior to penetration peg emergence (Gupta et al., 2015). Previously in *Cryptococcus neoformans*, it was also suggested that different virulence factors use different secretory pathways, such as through exosomes (see below) (Panepinto et al., 2009). Furthermore, the repression of *Sec6* and *Sec15* in the human pathogen *C. albicans* leads to a reduced secretion of aspartyl proteases and lipases involved in virulence (Chavez-Dozal et al., 2015a,b). However, the reduced macrophage death produced by the *Sec6* mutant may also be a consequence of its defect in filamentation and hyphal branching (Chavez-Dozal et al., 2015a).

During infection of cucumber with *Colletotrichum orbiculare*, fluorescently labeled effectors are actively accumulated in a ring-like structure which resembles the *M. oryzae* BIC (Irieda et al., 2014). In the same study, Irieda et al. observed that the delivery of these effectors is Sec4- and Sec22-dependent and, as a consequence, the loss of either of these two proteins causes a defect in virulence (Irieda et al., 2014).

These findings could indicate that fungal effectors are delivered to the host through different secretion routes involving distinct exocyst subunits but that the secretory pathway may be conserved in pathogenic fungi with similar infection strategies.

## The exocyst complex in plants

### The “green” exocyst complex

The exocyst complex exists in higher plants. This is true for dicotyledonous (“broad leaf”) and monocotyledonous (“grass”) plants that include key cereal crops such as rice and wheat. All exocyst components identified in metazoans and fungi have been conserved in plants. However, most subunits exist as paralogs. In the model plant *Arabidopsis thaliana* there are commonly two isoforms for a single subunit (SEC3, SEC5, SEC10, and SEC15) increasing to three paralogs for Exo84 and to over 20 for Exo70 (Elias et al., 2003; Synek et al., 2006; Hala et al., 2008). Whole-genome duplications have occurred frequently during higher plant speciation and this has generated closely related sub-families of proteins (Maere et al., 2005; Synek et al., 2006). The functional redundancy between isoforms further complicates genetic analyses. Many of the most severe mutant alleles show reduced transmission in haploid gametes when alone or combined with mutant alleles of other complex members (Synek et al., 2006; Hala et al., 2008). Moreover, mutation of the two *A. thaliana* SEC3 loci (that contain two SEC3 isoforms) results in embryo lethality (Zhang et al., 2013). The vast majority of plant exocyst functional data come from heritable loss-of-function alleles and not from conditional or non-heritable phenotypes. Any allele that is lethal in the haploid state would not be transmitted in either germline. This is unlike the situation in fungi where conditional alleles have been used extensively for functional analyses. It is therefore possible that all components of the exocyst are critical for plant life even though there is only evidence from selected subunits. The use of CRISPR/Cas9 mediated gene targeting (Hyun et al., 2015), inducible RNA interference (RNAi) approaches effective against essential genes (e.g., Ketelaar et al., 2004) and the identification of the drug endosidin 2 (Zhang et al., 2016) are likely to further expand the range of plant exocyst phenotypes. Recently, tissue-specific expression of RNAi for each exocyst subunit was used to demonstrate that the exocyst is essential for Arabidopsis fertilization (Safavian et al., 2015), elegantly demonstrating the use of this technique to circumvent lethal phenotypes.

The body of evidence for these individual components functioning as a complex is convincing (Zarsky et al., 2013). Binary interactions and partial-complex purification are consistent with conservation of “canonical” core subunit interactions. Five interactions between complex members have been confirmed through co-immunoprecipitation from plant tissue and nine through yeast 2-hybrid (Hala et al., 2008; Fendrych et al., 2010; Zarsky et al., 2013). These interaction data are broadly in line with the recent work exploring the structure of the *S. cerevisiae* complex (Heider et al., 2016). Data describing the structural biology of the plant exocyst is extremely limited. Interpretations of plant exocyst architecture are built upon primary sequence conservation in key structural domains, and supported by secondary structure prediction (Zarsky et al., 2013). Cross-kingdom complementation experiments have not been reported for plant exocyst components. However, a plant Exo70 has been found to have activity in a mammalian cell type (Ding et al., 2014), although Exo70 is known to exert effects on membranes in isolation from other complex members (Zhao et al., 2013).

Visual co-localization of exocyst components *in planta* has generated a variety of endomembrane localization patterns that suggests a complex and dynamic interplay between the subunits. Much of this variety could however be due to isoform-specific, tissue-specific and label-induced effects. Each plant tissue appears to have a distinct combination of paralog expression (Hala et al., 2008; Li et al., 2010), thus creating unique opportunities for component distribution and interactions. Further complication arises from stimulus-responsive expression of some paralogs (Chong et al., 2010; Pecenkova et al., 2011). At least four *A. thaliana* fluorescent protein fusions used to localize the exocyst have been shown to complement their respective mutant phenotypes (SEC3a, Exo84b, SEC8, and Exo70B1; Fendrych et al., 2010; Zhang et al., 2013) and the majority of observations using these probes underpin the familiar dogma that the plant exocyst is recruited to the PM. An exception is Exo70B1. This fusion protein complements its cognate mutant phenotype but is recruited to autophagosomes where Exo70B1 contributes to efficient autophagy (Kulich et al., 2013). Immunofluorescence of fixed pollen tubes associates endogenous SEC6, SEC8, and Exo70A1 to a shared population of endomembrane compartments at the growing tip and to the PM of the tube (Hala et al., 2008). Pollen tubes are cells that grow extremely rapidly through tip-focused secretion and are exploited as a model tissue for understanding plant cell polarity and exocytosis. Taken together these experiments support the existence of a plant exocyst complex that functions in a broadly analogous manner to its fungal and metazoan counterparts.

### The trafficking role(s) of the plant exocyst

**Figure 2:**
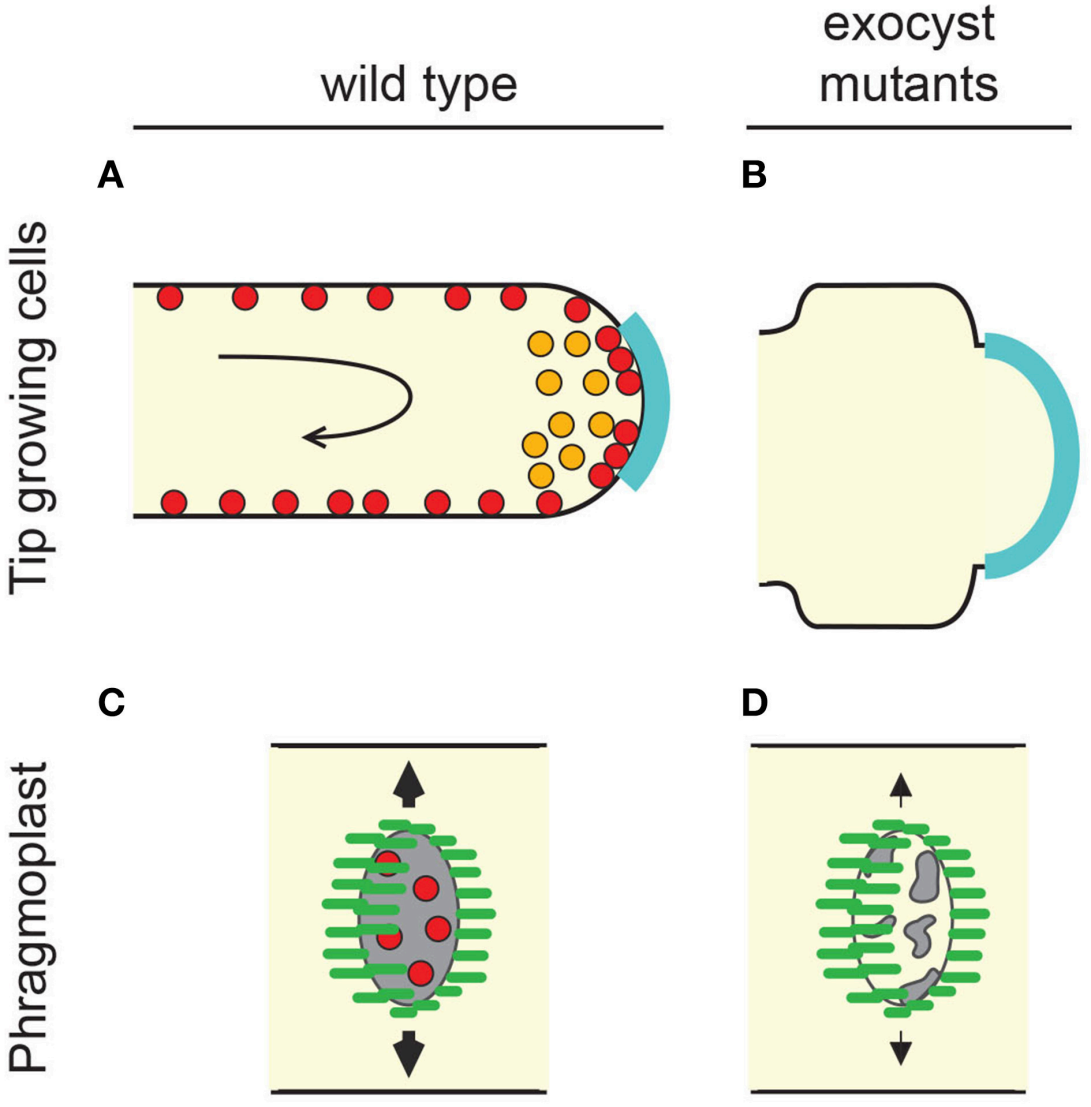
**The plant exocyst supports tip-growth and cell division. (A)** Tip-growing cells in plants (pollen tubes and root hairs) incorporate membrane and other cargoes delivered via vesicles to the apical growth zone of the PM (cyan). Rapid, rotational streaming of cytoplasm (black arrow) occurs behind the apex and is driven by myosin and actin cables. In pollen tubes SEC6, SEC8, and Exo70 label endomembrane compartments within the apex (orange). In both pollen tubes and root hairs exocyst components decorate the PM in a punctate pattern (red). **(B)** Loss of exocyst causes shortening and broadening of tip-growing cells. This is potentially consistent with a hypothetical widening of the growth zone (cyan) but could also be caused by alterations in cell wall properties that could result in turgor-driven expansion. **(C)** Plant cells produce new cell plates (gray) through the action of a cytoskeletal array known as the phragmoplast (microtubule organization within the phragmoplast is depicted in green). The phragmoplast expands from the former site of the spindle and advances toward the outer cell walls where the cell plate will eventually fuse. Exocyst components are enriched at the developing plate during two distinct plate growth phases (red) (Fendrych et al., 2010; Zhang et al., 2013; Rybak et al., 2014). **(D)** Exocyst mutants show a variety of cell plate phenotypes including reduction in the rate of cell plate expansion, increase in the probability of cell plate collapse, and alteration of the content of developing cell walls. See text for details and other references.

Cytokinesis in plants requires the building of a new cell wall between two daughter cells. This is orchestrated by a cytoskeletal array unique to plants named the phragmoplast. The phragmoplast is derived from the disassembly of the anaphase spindle with the purpose of guiding a cloud of vesicular material to the site of cell plate construction (Figure 2C). The phenotypes of *A. thaliana* Exo84 isoform “b” and the SEC3 locus support the proposal that the exocyst is necessary for organized cytokinesis and cell plate maturation (Fendrych et al., 2010; Zhang et al., 2013) (Figure 2D). 3.5% of cells mutant for Exo84b showed evidence of failed cytokinesis (Fendrych et al., 2010) while SEC3 mutants showed “normal” cell plate maturation but no organization of cell plate orientation, causing early embryo arrest (Zhang et al., 2013). Close observation of Exo70 isoform “A1” mutants has also revealed a subtle defect in the process of cell plate assembly (Fendrych et al., 2010). The process of cell plate deposition is performed in close co-operation with the TRAPPII membrane-tethering complex. This is largely a sequential division of responsibilities during the maturation process but co-immunoprecipitation experiments suggest the possibility of physical bridging between the TRAPPII and exocyst complexes (Rybak et al., 2014).

Work on cell plates of dividing cells and the tips of pollen tubes have demonstrated bulk recruitment of exocyst subunits but the crowding of secretory machinery at these structures prevents the observation of recruitment *sequences*. Two studies have utilized interphase cells to observe “real-time” exocyst dynamics (at rapid frame acquisition rates) across relatively large areas of PM and cell cortex (Fendrych et al., 2013; Zhang et al., 2013). Both studies showed that exocyst components are recruited transiently as punctate structures that can be resolved using diffraction-limited fluorescence microscopy. SEC3A-GFP punctae exist at densities of 0.4–0.6 /μm^2^ and appear immobile (Zhang et al., 2013). These patches exist for a median time of only 4 s before disappearing from the membrane. This is likely through a process of patch disassembly as no punctate structures decorated with SEC3-GFP can be identified using spinning disk confocal microscopy within the cytoplasm (Zhang et al., 2013). Fluorescent protein fusions to SEC6, SEC8, Exo70A1, and Exo84b are also distributed in punctae at the PM and have been recorded at higher densities of 1.2–1.6/ μm^2^ (Fendrych et al., 2013). Co-localization analysis confirmed that over a third of punctae shared binary combinations of SEC6, SEC8, and Exo84b. These binary combinations arrived and departed in synchrony with a residency time of over 10 s; significantly longer than the SEC3-GFP study. A vesicle-resident SNARE protein (VAMP721) was found to co-occupy a small but statistically significant number of Exo84b patches suggesting that exocyst punctae can coincide with vesicles pausing at the PM (Fendrych et al., 2013). Co-imaging of SEC3A and further exocyst components is urgently required to consolidate the observations but a model is emerging where the exocyst assembles, or arrives in a preassembled state, to exist as a transient, anchored nano-structure at the PM that can interface with stationary vesicles. Equally these exocyst patches/particles are capable of completing their residency at the cell cortex without the presence of a vesicle, possibly suggesting that the plant exocyst provides windows of opportunity for PM-vesicle interaction and fusion (Fendrych et al., 2013; Zhang et al., 2013).

Any discussion of the molecular function of the plant exocyst must include the diversity of the plant Exo70 subunit. This gene family has shown broad diversification linked to functional specification. There are 23 Exo70 genes within the *A. thaliana* genome and these consist of a series of fundamental subclasses that are conserved across higher plants and are named alphabetically from A to I (Synek et al., 2006). The functional specialization of individual Exo70 isoforms has been demonstrated experimentally. Exo70A1 of *A. thaliana* is widely expressed with mutant phenotypes that suggest this isoform has a basal secretory function for cell wall and cuticle development in multiple cell types (Fendrych et al., 2010; Kulich et al., 2010; Li et al., 2013). Moreover, the function of this isoform has an impact upon organ development through the plant hormone auxin. The trafficking of auxin efflux proteins is inefficient in *exo70a1* mutants. This causes an abnormal distribution of the hormone and perturbs signaling processes that guide root growth (Drdova et al., 2013). The *A. thaliana* Exo70B1 isoform is required for autophagy and delivery of endomembrane compartments to the vacuole (Kulich et al., 2013). This transport pathway plays a critical role in receptor signaling during plant immune responses and the closely related Exo70B2 isoform has a pathogen defense phenotype (Pecenkova et al., 2011). Exo70C1 contributes to pollen tube growth (Li et al., 2010). Exo70E2 promotes the formation of double membrane secretory organelles (Ding et al., 2014) and Exo70H4 is required for callose deposition (a carbohydrate cell wall polymer) in specific cell types (Kulich et al., 2015).

The properties of specific Exo70 variants are conserved across kingdoms. Exo70 isoforms capable of forming exosomes destined for unconventional secretion can do so effectively in mammalian cells (Ding et al., 2014). This suggests exciting opportunities for endomembrane manipulation through heterologous expression of novel Exo70 isoforms. It also postulates a model where the general membrane tethering and fusion-regulating properties of the exocyst complex can be directed using specific Exo70 “warheads.” The cells of higher plants place unusual demands on secretion machinery, requiring cargoes to be sorted within a thin layer of cytoplasm to distinct destinations spread over a vast cell surface. The proliferation of Exo70 may have occurred in part in response to this demand for overlapping yet spatially and temporally distinct routes for secretory traffic.

### Making a point—a functional comparison of the plant and fungal exocyst

A comparative analysis of exocyst function across eukaryote kingdoms is perhaps best performed in tip-growing plant cells. These are analogous in their growth style to fungal hyphae and some membrane protrusions of animals and protozoa. Most plant cells are considered to expand using “intercalary” growth where new cell wall material and new PM are integrated across relatively wide areas. Tip-growing cells are less common but include pollen tubes (the structures that emerge from pollen grains and deliver sperm nuclei to oocytes) and root hairs (long tubular extensions of root epidermal cells that anchor roots and provide surface area for water and nutrient uptake). In these cells exocytosis is focused, but not necessarily exclusive, to the very tip of the cellular extension (Rounds and Bezanilla, 2013). Thick actin bundles and myosin-mediated motor action dominate transport processes throughout most of the cytoplasm of tip-growing cells but toward the tip the actin cytoskeleton is frayed into finer, dynamic filamentous arrays that are essential for cargo integration at the cell cortex. The details of this arrangement differ between pollen tubes and root hairs but in both cases bulk endomembrane compartment transport does not extend fully to the tip where the cytoplasm is enriched with exocytic and endocytic endomembrane compartments. There is no defined analog of the SPK.

Components of the exocyst have been identified at the apex of tip-growing cells (Figure 2A). Immunofluorescence using antibodies raised to SEC6, SEC8, and Exo70A1 show these components located within the secretory vesicle-mass at the tip of pollen tubes (Hala et al., 2008). The same study identified genes encoding four subunits as being significant contributors to pollen tube growth (SEC5, SEC6, SEC8, and SEC15). Mutant tubes showed a tendency for increased width and reduced length (Figure 2B). These features are indicators of a loss of growth polarity that are also associated with aberrant small GTPase signaling and impaired actin dynamics. A forward genetics screen in maize identified *roothairless1* (*rth1*), a mutant where root hairs initiate but do not elongate. Cloning of the gene revealed it to be an isoform of SEC3 (Wen et al., 2005). Surprisingly, despite the catastrophic impact on root hair tip-growth, pollen tube function appears normal (Wen et al., 2005). This may indicate genetic redundancy with other SEC3 paralogs. In *A. thaliana* loss-of-function alleles of SEC3a have proven to be embryo lethal (Zhang et al., 2013) but transmission through the male germline is again unaffected. Unlike exocyst mutants of neurons and some filamentous fungi, no pollen tube branching phenotypes have been reported from plant exocyst mutants. An allele of Exo70A1 does however cause an increase in root hair branching from wild-type levels of 22–56% (Synek et al., 2006).

Live-cell imaging of SEC3a-GFP in root hairs has shown no enrichment at the tip. Instead the protein decorates the PM in a punctate pattern but at an even intensity along the length of the growing root hair with only a 30% enrichment at the tip (Figure 2A) (Zhang et al., 2013). It is not known whether this behavior is specific to SEC3a or, alternatively, whether other exocyst components are localized in a pattern more analogous to that observed in pollen tubes. It is interesting to note that SEC6, SEC8, and Exo70A1 also decorate the PM of pollen tubes in a punctate pattern when observed beyond the tip-growth zone (Hala et al., 2008). In summary, exocyst components are essential for tip growth in plants but they are not necessarily precise markers for indicating sites of tip-polarized exocytosis.

Fungal tip growth is distinctly different. Long-distance transport in fungal hyphae, like in mammalian cells utilizes bi-directional movement on MTs rather than unidirectional transport on actin cables. The exchange between distribution systems occurs in the zone of the SPK, so an absence of “long range” to “short range” transference may also explain the lack of a plant SPK analog. Equivalent endomembrane compartments do accumulate at the tip of plant cells but the borders of these masses are less defined. The distribution of plant exocyst components in tip growing cells is, in general, less focused to the polar site of cargo delivery in comparison to fungal hyphae. “Foci” of exocyst components appear transiently at the plant PM for periods of 4–10 s *throughout* the tip-growing cell, yet paradoxically the complex is required for tip-focused secretion. One speculative interpretation is that the cross-kingdom comparison suggests that plant exocyst localization is not necessarily indicative of an *active* exocyst population. This is in contrast to the fungal exocyst where localization is tightly correlated with activity.

### The exocyst and plant disease

Very recently the plant exocyst has emerged as a potential key player in plant-pathogen interactions. The evidence for this can be grouped into three streams: 1/mutant phenotypes that reduce basal immune defenses; 2/sub-cellular localizations associated with microbe contact; 3/gene-for-gene interactions that suggest exocyst components are targets of pathogen effector proteins. The most direct evidence is the impact upon microbial interactions caused by the loss of function of the host exocyst. This has not been explored in a methodical fashion using all exocyst subunits but two Exo70 isoforms of *A. thaliana* were identified as being expressed in response to pathogen challenge (Pecenkova et al., 2011). Loss of function phenotypes of these two genes include increased susceptibility to the bacterial phytopathogen *Pseudomonas syringae* and aberrant defense structures in response to powdery mildew (Pecenkova et al., 2011). Pathogen *perception* appears to be a key role for Exo70B2. Disruption of Exo70B2 activity perturbs the function of receptors that respond to microbial molecular patterns such as flagellin and chitin (Pecenkova et al., 2011; Stegmann et al., 2012). Defense responses are not activated efficiently without Exo70B2 function. These relationships between microbes and the exocyst could be important in an agricultural context. Transient gene silencing assays revealed that Exo70F function supports defense against powdery mildews in barley (Ostertag et al., 2013). This relationship is likely to extend to other closely related cereal crops such as wheat, rice and maize.

Many plant-microbe interactions are beneficial for plant life. Arbuscular mycorrhizas are root-fungal interactions that are promoted by reciprocal signaling between host and microbe. The plant benefits through increased efficiency of nutrient absorption and this process requires the development of specialized interfaces between plant and fungal cells. In *Medicago truncatula* multiple markers of exocytosis are recruited to these interfaces including Exo84 and Exo70I (Genre et al., 2012; Zhang et al., 2015). Exo70I function is critical for the formation of the interface and in its absence fungal growth into host cells is restricted and markers indicative of interface membrane identity are depleted. Remodeling of membranes to accommodate microbes therefore appears to be a role for the host exocyst complex.

Evidence is accumulating that multiple classes of phytopathogens have developed adaptations that specifically suppress or manipulate the host exocyst complex. The rice pathogen *M. oryzae* has a suite of secreted proteins to suppress host defense that includes the protein AVR-Pii. Co-immunoprecipitation from rice cells using this protein as bait isolated the Exo70 isoforms Exo70F2 and Exo70F3 (Fujisaki et al., 2015). A rice gene present in some cultivars (called Pii) can confer resistance to *M. oryzae* by responding to the presence of AVR-Pii. The rice Pii gene encodes a cytosolic receptor for AVR-Pii. Rice Exo70-F3 is required for *M. oryzae* AVR-Pii to trigger a response from rice Pii (Fujisaki et al., 2015). One proposed explanation for this is that Pii is “guarding” Exo70 and responds when pathogen effectors disrupt this interaction. These relationships are proving to be common in gene-for-gene defense responses. An analogous scenario has been described in *A. thaliana* powdery mildew interactions (Zhao et al., 2015). Here the host Exo70 isoform is Exo70B1 and the receptor is TIR-NBS2. Loss of Exo70B1 disrupts the interaction between the two host proteins and leads to constitutive activation of immune responses. Phytopathogenic oomycetes also target exocyst components. The *Phytophthora infestans* (potato blight) effector AVR1 binds SEC5 to disrupt secretion and programmed cell death responses (Du et al., 2015). These relationships infer that the host exocyst is a significant battleground in plant disease progression. Excitingly many of these examples are derived directly from critical crop plants and their agriculturally damaging pathogens. This opens the potential for immune system reinforcement, based around engineering of the exocyst complex, that will have high-value in safeguarding world food supplies.

## The exocyst complex in metazoa

The mammalian exocyst complex also contains eight sub-units that are ubiquitously expressed. They are often called by their Sec or Exo names for simplicity, but are officially labeled EXOC1 (= Sec3), EXOC2 (= Sec5), EXOC3 (= Sec6), EXOC4 (= Sec8), EXOC5 (= Sec10), EXOC6 (= Sec15), EXOC7 (= Exo70), and EXOC8 (= Exo84). In mammals, each sub-unit has several isoforms produced by alternative splicing (UniProt Consortium, 2015). In the absence of systematic spatio-temporal analysis, the significance of this diversification is at present largely unknown.

### The exocyst complex during development

Existing full knockout (KO) mice of members of the exocyst show early embryonic lethality, from the blastocyst stage. The blastocyst forms when 64 cells differentiate into 2 lineages: the inner cell mass (ICM), which will give rise to the embryo; and a peripheral layer of cells, the trophoblast which will give rise to the placenta and mediate the attachment of the embryo to the uterus wall. The blastocyst then implants in the uterus through its trophoblast. The later gastrula reorganizes as an ectoderm, mesoderm, and endoderm that will subsequently develop into tissues and organs. *Sec3*^−/−^ embryos have a peri-implantation lethal phenotype, due to defects in ICM proliferation (Mizuno et al., 2015). *Sec8* null homozygous mutant mice initiate gastrulation but show a defect in mesoderm formation (Friedrich et al., 1997). Other conditional, partial, or heterozygous mutant mice are characterized by a range of tissue-specific developmental defects. A conditional Sec10 KO mouse exhibits defects in the organogenesis of its genito-urinary track. It develops a type of chronic kidney disease characterized by the obstruction of the urinary tract. This leads to complete anuria in newborns, with death occurring 6–14 h after birth (Fogelgren et al., 2015). Sec15 is involved in late erythroid differentiation in the bone marrow (Bloom and Simon-Stoos, 1997). Mice in which *Sec15* is truncated (aka *hbd*, hemoglobin-deficit mouse) fail to accumulate iron in their reticulocytes and have small reticulocytes (Lim et al., 2005; White et al., 2005; Garrick and Garrick, 2007). The phenotype can be rescued by transplantation of normal bone marrow and transplantation of a sick bone marrow into healthy mice gives rise to a *hbd* phenotype. Bizarrely, these *Sec15* mutant mice have no other phenotypic defects despite the fact the protein is expressed in other, non-hematopoietic tissues (Lim et al., 2005).

The exocyst is involved in the development of the placenta from the blastocyst trophoblast. The syncitiotrophoblast is a non-proliferative layer of the trophobtast. It is directly in contact with the mother blood and facilitates nutrient and gas exchange between mother and fetus. It also secretes signaling hormones and lytic enzymes that cause apoptosis of the epithelium of the uterus, necessary for its implantation. Some subunits of the exocyst complex are present in the apical membrane of the human syncytiotrophoblast (Vandre et al., 2012; Gonzalez et al., 2014). ExoC1-6 show a punctuate signal throughout the cytosplasm, whereas Exo70 and Exo84 are also enriched at or near the apical PM. It was suggested that alterations in exocyst function may be associated with preclampsia, a trophoblastic condition that leads to poor placental vascularization.

It is probable that the ubiquitous expression and multiple functions of the exocyst complex that we develop below are the source of these dramatic and early embryonic phenotypes.

### The exocyst complex arborizes neurons

Neurons are highly polarized cells, typically made of one long axon and several shorter, arborized dendrites. These neurites branch out from a cell body and extend to form connections with neighboring cells and ultimately establish complex circuits. Dendrites typically function to receive signals while axons send signals to neighboring cells. At the tip of the axon, a specialized type of exocytosis, called synaptic transmission regulates neurotransmitter release. Surprisingly, the exocyst is not involved in the tethering of these specialized vesicles (Murthy et al., 2003). It was suggested that the exocyst marks the sites of future synapse formation but plays no role in the mature synapse (Hazuka et al., 1999). This hypothesis is challenged by the fact Sec6 was observed at the PM of mature synaptic terminals (Vik-Mo et al., 2003). It is now apparent that the exocyst is present at multiple sub-cellular locations, which raises the question of its role(s) in neuron biology.

**Figure 3:**
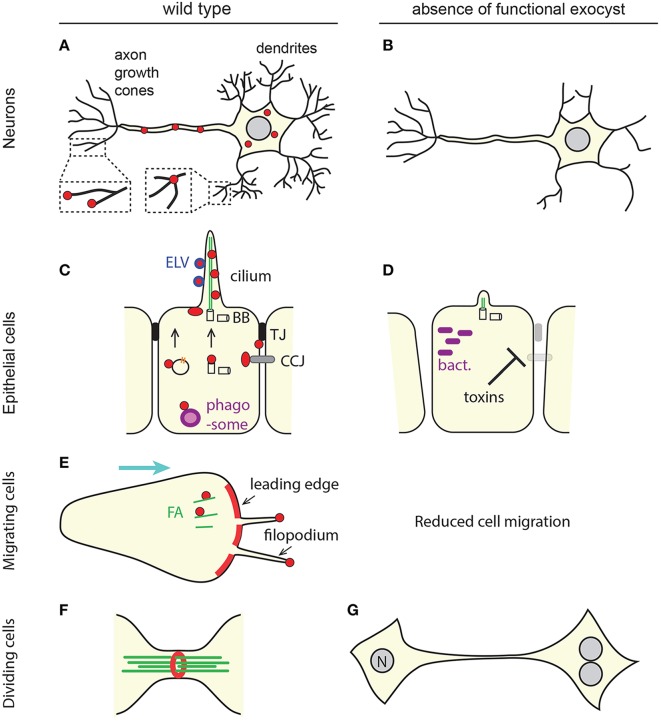
**Some of the multiple roles of the animal exocyst complex. (A,C,E,F)** Overall localization of the exocyst in physiological conditions (red). For simplicity, the differential localization of individual sub-units is not represented here. The exocyst is mostly present at zones of bulk (leading edge, **E**), or “finger-like” (neurites, **A**; cilia, **C**; filopodia, **E**) membrane extension. **(A)** In neurons, some subunits accumulate at dentritic branching points, but not at growing tips of dendrites, whereas others localize to the tip of axon filopodia. **(C,E)** The exocyst localizes at sites of cell-cell or cell-matrix junctions (tight junctions, TJ, black; cell-cell junctions, CCJ, gray; focal adhesions, FA, green). It was also reported to be associated with trafficking vesicles carrying cell surface receptors (orange), with the migrating basal body (BB), with phagosomes or phagosome-fusing endosomes (purple) and with extra-cellular exosome-like vesicles (ELV, blue). **(F)** The exocyst can organize as a ring. **(B,D,G)** Cellular phenotypes associates with a loss of exocyst function. Protrusions necessary for cell function or migration are not formed, cell polarity and tissue integrity are compromised, phagosome acidification is altered which constitutes a window of opportunity for pathogenic bacteria, cells fail to complete cytokinesis, and undergo extra mitoses (nucleus, N). **(D)** Some toxins secreted by bacteria impair the delivery of the exocyst at cell junctions. See text for details and references.

In drosophila, the development of the optic circuits is well documented and it is possible to know if a photoreceptor neuron reaches its target partner neuron at the right ganglion. *sec15*^−/−^ neurons extend normally and form normal synapses but fail to select their right synaptic partner (Mehta et al., 2005). At the distal tip of extending neurites, growth cones probe the environment and guide neurite outgrowth. Growth cones are very dynamic structures that continuously extend and retract actin-rich membrane protrusions such as filopodia and lamellipodia. Axons and dendrites outgrowth therefore relies on membrane expansion and cytoskeletal remodeling. Highlighting the prominent role played by the exocyst in the determination of neuronal cell polarity, neurite outgrowth is impaired in the absence of functional exocyst subunits in various systems such as primary neurons, cultured PC12 cells or multicellular model organisms (Figure 3B) (Vega and Hsu, 2001; Murthy et al., 2003; Lalli, 2009; Das et al., 2014; Peng et al., 2015). This phenotype further accounts for the role of the exocyst during neuroblast migration and brain development (Letinic et al., 2009; Das et al., 2014).

Axon and dendrite outgrowth may however rely on different mechanisms. In developing hippocampal neurons, the exocyst is highly enriched in axon growth cones and in filopodia, where it probably participates in membrane extension by mediating the fusion of plasmalemmal precursor vesicles with the PM, at the most distal end of the axon (Figure 3A) (Hazuka et al., 1999; Dupraz et al., 2009). The exocyst may also orchestrate actin organization within filopodia (Hertzog and Chavrier, 2011) (see below). In mice skin melanoma cells, Exo70 induces filopodia formation through its ability to deform membranes (Zhao et al., 2013) and it is possible that Exo70 plays a similar function in neurite outgrowth. At the membrane of the synapse, the exocyst may recruit and act as a scaffold for plasticity related proteins, such as post synaptic density 95 (PSD-95) (Riefler et al., 2003). A scaffolding role was also suggested during starvation- and pathogen-induced autophagy, where the exocyst mediates the assembly and activation of the autophagy machinery (Bodemann et al., 2011).

Unlike axons, terminal dendrites may not grow by direct tip extension or rely on distal, targeted exocytosis (Peng et al., 2015). Instead, the source of material for terminal dendrite outgrowth is thought to be provided by a unidirectional lateral diffusion from primary dendrites. Exocytosis and the exocyst are not at the distal tips of dendrites but concentrate at dendritic branches, from where they orchestrate dendritic arborization (Figure 3A) (Peng et al., 2015; Taylor et al., 2015; Zou et al., 2015). In *C. elegans* PVD neurons- a highly branched network of sensory neurons that envelope the worm and regulate its body posture and movement (Albeg et al., 2011), loss of function mutants of *exoc-8* and *sec-8* cause dendritic arborization defects, due to the fact pro-branching trans-membrane proteins are not delivered to the proximal dendritic membranes (Zou et al., 2015). A role of the exocyst in arborization rather than tip growth was also reported in drosophila tracheal terminal cells and may therefore not be neuron-specific (Jones et al., 2014).

Several exocyst subunits interact in a Ral-dependent manner with the Par3–aPKC-Par6 complex (Partitioning-defective and atypical protein kinase C), a master regulator of polarity (Lalli, 2009). The exocyst-Par axis exists in other cell types besides neurons, where it similarly regulates polarity (e.g., Rosse et al., 2009; Zuo et al., 2009; Bryant et al., 2010). It appears that the exocyst targets Par-aPKC to polarized zones and conversely aPKC actively regulates the polarized delivery of the exocyst (Rosse et al., 2009). As well as targeting polarity factors at sites of polarized growth, the exocyst also regulates the delivery of receptors. Exo70 is required to insert the IGF-1 receptor into the membrane of the growth cone, which stimulates axon elongation and membrane expansion through the Rho GTPase TC10 (Dupraz et al., 2009; Fujita et al., 2013).

The long distance transportation of exocyst subunits along the axon is mediated by MTs, which explains that Sec6 and Sec8 were immunolocalized to the cell body and axon and that the exocyst was co-immunoprecipitated with MTs in rat brain lysates (Vega and Hsu, 2001). However, there is to date no reported evidence that the exocyst complex directly regulates the dynamics or organization of MTs like it does with the actin cytoskeleton.

### The exocyst complex establishes epithelial polarity, morphogenesis, and homeostasis

The role of the exocyst complex in polarization is further exemplified in epithelial apico-basal polarity (Blankenship et al., 2007). Epithelial cells are held together and to the matrix through specialized junctions that help orient the resulting tissue. Adherens junctions and desmosomes are type of cell-cell junctions, in which adhesion molecules on opposing cells interact and bridge cytoskeletal component of neighboring cells. Upon initiation of cadherin-mediated cell-cell adhesion, the exocyst relocates from the cytoplasm to sites of cell-cell contacts, where it interacts with junctional proteins (Grindstaff et al., 1998; Yeaman et al., 2004). In an invasive cell line that expresses no cadherin, the exocyst is however no longer localized to sites of cell-cell contacts, but instead relocates to protrusions that preclude cell migration (Spiczka and Yeaman, 2008) (Figures 3C,E). Conversely, the exocyst is involved in efficient targeting of these adhesion molecules. Interactions between type I phosphatidylinositol-4-phosphate 5-kinases and Exo70 governs the binding of the epithelial cadherin E-cadherin. Knockdown (KD) of Exo70 perturbs correct clustering of E-cadherin at adherens junctions and KD of Sec3 causes inefficient targeting of desmosomes (Andersen and Yeaman, 2010; Xiong et al., 2012). In certain growth conditions, epithelial Madin-Darby canine kidney cells (MDCK) organize as a hollow cyst and if stimulated, can extend tubules from the basolateral surface of individual cells. In non-polarized MDCK cells, the exocyst is cytosolic but in polarized cyst cells and mature tubule cells, it localizes to tight junctions (Lipschutz et al., 2000) (Figure 3C). Tight junctions seal epithelial cells and play two functions: they limit the passage of small molecules between cells and they block lateral diffusion within the lipid bilayers. They are therefore critical to maintain an apical and a basolateral domain and are a central platform for regulation by the Par complex with which the exocyst collaborates. Accordingly, over-expression of Sec10 in MDCK cells results in increased tubulogenesis due to an increased delivery of baso-lateral proteins (Lipschutz et al., 2000).

The role of the exocyst complex in epithelium polarity has consequences for embryonic development. In fly embryos, cellularization is the process by which a large syncytium containing thousands of nuclei is subdivided into separate cells. Sec5 mediates cellularization by directing the addition of new membranes from the apical end of the lateral membranes (Murthy et al., 2010).

A function of epithelia is to create a physical barrier between the external milieu and the body interior. Because it strengthens cell-cell junctions, the exocyst protects epithelium barrier integrity (Figure 3D). Cholera toxin weakens intercellular bonds at the basolateral surface of the intestinal epithelium, by inhibiting the Rab11/exocyst-mediating trafficking of E-cadherins to adherens junctions. This leads to a massive efflux of water across the intestinal epithelium, characteristic of the water diarrhea observed in patients infected with *Vibrio cholera* (Guichard et al., 2013). Similarly, two toxins secreted by *Bacillus antracis* target Rab11/Sec15, affect the formation and signaling at adherens junctions and lead to the disruption of the endothelial barrier and the lethal vascular leakage associated with systemic anthrax (Guichard et al., 2010).

Another protective function of the exocyst complex may come from its ability to somehow regulate endocytosis in metazoan like it does in yeast (Oztan et al., 2007; Jourdain et al., 2012; Jose et al., 2015). Sec10 protects kidney epithelium integrity against oxidative stress associated with kidney ischemia and reperfusion (I/R) injury, by increasing EGF receptor endocytosis and signaling, and downstream activation of the MAPK pathway (Park et al., 2010; Fogelgren et al., 2014). Sec10 may also be involved in kidney recovery after I/R injury, which may have implications for transplantation and vascular surgery (Park et al., 2010).

The exocyst participates in cell adhesion with the substratum. Silencing of Exo70 by siRNA inhibits cell spreading on fibronectin-coated substratum (Hertzog et al., 2012). On the one hand, the exocyst participates in the RalA-dependent delivery of focal adhesion proteins such as integrins at the leading edge of migrating cells (Balasubramanian et al., 2010; Thapa et al., 2012). Cell adhesion to the substratum is a prerequisite to cell migration and interfering with exocyst function leads to cell motility defects (Spiczka and Yeaman, 2008). Accordingly, some, but not all exocyst subunits colocalizes and co-fractionates with paxillin-containing focal complexes in pseudopods of migrating cancer cells (Spiczka and Yeaman, 2008) (Figure 3E). Neuron guidance defects observed in *Sec15*^−/−^ drosophila photoreceptors are also associated with defects in the trafficking of adhesion molecules (Mehta et al., 2005). On the other hand, upon cell detachment from the substratum and re-adhesion, Exo70 regulates the recycling of the mechanosensing and endocytic protein caveolin-1 to the PM, away from focal adhesions (Hertzog et al., 2012). Opposite from its role as a tether or scaffold, the exocyst may therefore also mediate the exclusion of factors from specialized PM domains.

### Does the exocyst complex interact with the centrosome?

Disruption of the exocyst complex affects cytokinesis in animal cells and yeast in a similar manner (Wang et al., 2002; Echard et al., 2004; VerPlank and Li, 2005; Neto et al., 2013a; Giansanti et al., 2015). In both cases, cytokinesis involves the formation of a medial actin ring, which, upon constriction, drags the PM. Animal cytokinesis does not involve septum formation and further differs from yeast cytokinesis by the fact that the ingressed cleavage furrow leaves a narrow cytoplasmic bridge between daughter cells at the end of cytokinesis. This midbody is made of overlapping MTs and many other proteins whose function is to define the site of abscission. In drosophila the exocyst complex localizes at the cleavage furrow and is involved in the early stages of furrow ingression (Giansanti et al., 2015). Later, it localizes at the site of abscission (Skop et al., 2004). More specifically, the exocyst forms a midbody ring through its interaction with centriolin, a protein classically associated with centrosome maturation (Gromley et al., 2005) (Figure 3F). Similar to loss of centrosome function, loss of exocyst function leads to the accumulation of several multinucleated cells connected by long intracellular bridges that went through a second mitosis without having divided (Chen et al., 2006) (Figure 3G). These findings support the notion of a putative collaboration between the centrosome or centrosome-associated proteins and the exocyst complex. This is strengthened by the observation that centrosome and endosomes interact (Hehnly et al., 2012). The exocyst also tethers recycling endosomes but it is unknown whether this is in connection with its interaction with centrosome proteins at the midbody (Fielding et al., 2005; Chen et al., 2006; Hehnly et al., 2012; Neto et al., 2013a). The discovery that centriolin/exocyst connects the two organelles may shed light on the functional significance of this association. The implications go beyond the understanding of cell division, as both centrosomes and membrane-bound vesicles are involved in other cellular processes such as polarized migration and ciliogenesis.

### Exocyst genes are ciliopathy genes

Most mammalian cells build primary cilia, sensory organelles that act as chemo- or mechanosensors and transducers of signals that regulate key developmental signaling pathways. Primary cilia have a characteristic “9+0” architecture made up of nine doublet MTs arranged in a ring and surrounded by a ciliary membrane that is continuous with the PM. The primary cilium is extended from a basal body derived from one of the two centrioles of the cell's centrosome (Dawe et al., 2007). Ciliogenesis is a multi-stage process initiated by the translocation of the centrioles to the apical membrane. Centrioles then acquire two sets of distal and subdistal appendages (aka centriole maturation). During centrosome migration, a ciliary vesicle that is probably derived from the Golgi apparatus encapsulates the distal end of the mother centriole through association with the distal appendages (Sorokin, 1962; Schmidt et al., 2012; Joo et al., 2013). The exact mechanism behind centriole migration toward the apical cell surface is elusive but requires actin and the ciliary vesicle (Boisvieux-Ulrich et al., 1987, 1990; Lemullois et al., 1987, 1988; Pan et al., 2007; Park et al., 2008; Kim J. et al., 2010; Pitaval et al., 2010; Yan and Zhu, 2013; Kim et al., 2015). The ciliary vesicle fuses with the PM in a manner similar to exocytosis, thereby docking the centrioles into the cortical cytoskeleton (Ghossoub et al., 2011). After removal of a capping protein from its distal end, the mother centriole elongates an axoneme and the cilium is extended. Both rely on intraflagellar transport (IFT) (Scholey, 2003), a MT-directed trafficking mechanism that facilitates the bi-directional movement of cargoes between the cilium base and tip. Ciliary cargoes are necessary for cilia assembly, maintenance and signaling. IFT involves the recruitment and assembly of the IFT machinery with cargo at the cilium base and links the ciliary membrane to the basal body (Deane et al., 2001).

Besides a localization at tight junctions the exocyst complex also localizes at cilia in MDCK cells (Liu Q. et al., 2007). In fact, the exocyst complex displays several sub-ciliary locations (Figure 3C). Some exocyst sub-units were visualized at the base of the primary cilium (Rogers et al., 2004; Babbey et al., 2010; Seixas et al., 2016). There, the exocyst is believed to traffic and dock Golgi-derived vesicles carrying ciliary proteins. In support of this, Sec10 directly interacts with the ciliary proteins IFT20, IFT88, and polycystin-2, and the expression levels of some of these proteins depend on the level of expression of Sec10 (Zuo et al., 2009; Fogelgren et al., 2015). Moreover, the KD of *Sec10* in MDCK cells and of *Sec15* in a human retinal pigment epithelial cell line reduce cilium length, whereas the overexpression of Sec10 increases cilium length (Zuo et al., 2009; Feng et al., 2012) (Figure 3D). Antisense morpholinos of *sec10* in zebrafish produce curly tail up, left-right asymmetry defects, small eyes and oedema, that are characteristic of defective cilia (Fogelgren et al., 2015).

Some sub-units of the exocyst complex are also (e.g., Sec10) or solely (e.g., Exo70 and Exo84) present within the cilium itself (Feng et al., 2012; Chacon-Heszele et al., 2014; Seixas et al., 2016). This uniform distribution along cilia, but not at the cilium tip, is surprising and raises the question of the ciliary role of the exocyst. One possibility is that like in other organisms, ciliary vesicles actually exist that could carry exocyst proteins (Chacon-Heszele et al., 2014).

All members of the exocyst complex and most of its regulators, are also present in exosome-like vesicles (ELV), attached to the *outer* surface of cilia (Chacon-Heszele et al., 2014). ELV are released into the extracellular medium by budding of the PM at the ciliary base. They are considered a form of cell-cell communication, because they can bind to the neighboring cilium and release cilia-specific membrane proteins (Hogan et al., 2009; Wood et al., 2013). The ciliary matrix is continuous with the cytoplasm but at the transition zone, a physical barrier controls ciliary entry, and exit (Garcia-Gonzalo and Reiter, 2012). One hypothesis is that ELV are too large to pass this barrier but must be delivered to the nascent cilium of the same or adjacent cell as they contain proteins and lipids necessary for ciliogenesis. In this scenario, the exocyst would be involved in the release or uptake of ELV from and to cilia, or may unload or traffic taken up ELV cargoes along the cilium (Chacon-Heszele et al., 2014). This would explain the uniform localization of the exocyst within the cilium. Exosomes are not cilia-specific but the role of the exocyst in their biology may be (Hyenne et al., 2015). Fungi also secrete exosomes. In *C. neoformans*, Sec6 RNAi mutants are less virulent to mice than the WT because they cannot secrete exosomes and deliver virulence factors (Panepinto et al., 2009). It is possible that, like in epithelial cells, the exocyst complex controls exosome release by the fungus, or uptake by the host.

Another possible role of the exocyst complex may be in basal body positioning. Centriolin and cenexin/ODF2 are centriole appendage proteins and they mediate the association of the exocyst and some of its regulators with the mother centriole (Hehnly et al., 2012). Centrin on the basal body may bind the exocyst on the ciliary vesicle. In other words, the centrin-exocyst interaction bridges the basal body and ciliary vesicle (Park et al., 2008). In some respect this ciliary vesicle/centrosome interaction is similar to the endosome/centrosome interaction mentioned above (Hehnly et al., 2012). By targeting the ciliary vesicle toward the PM, the exocyst may therefore participate in the migration, planar positioning or docking of the basal body (Park et al., 2008; Huang and Lipschutz, 2014).

A number of inherited diseases, collectively termed ciliopathies, have been linked with defects in genes that affect cilium biogenesis or function (Waters and Beales, 2011). Ciliopathies share many clinical features, with kidney cysts, retinal degeneration and skeletal malformations often seen in combination with central nervous system developmental defects. The most severe are lethal in early gestation or shortly after birth. Exo84 was identified as a Joubert syndrome gene in a family originally diagnosed with an early onset neurodevelopmental disorder (Dixon-Salazar et al., 2012). Exome sequencing conducted on Arab families with Meckel-Gruber syndrome identified a single nucleotide pathogenic mutation in Sec8 (Shaheen et al., 2013). No other exocyst members have so far been associated with ciliopathies, probably because alterations are lethal. However, Sec10 genetically and physically interacts with the ciliary protein polycystin-2 whose mutation leads to autosomal dominant polycystic kidney disease (ADPKD), and knock down of Sec10 causes the generation of multiple kidney cysts with short primary cilia, a hallmark of ADPKD (Fogelgren et al., 2011; Seixas et al., 2016). Thus, two, possibly three, exocyst genes happen to be ciliopathy genes.

Many ciliary proteins now appear to have both ciliary and non-ciliary functions that may equally account for the cellular defects that yield ciliopathies. This is very likely to be the case for the exocyst, but it is clear that the complex has a role to play in cilium assembly and function.

### A role of the exocyst in long range cell-cell communication

Tunneling nanotubes (TNTs) are a type of intercellular communication that bridge two or more cells over long distances with no contact with a substratum (Rustom et al., 2004). They are used to shuttle material from cell to cell and to transduce molecular and electrical signals. TNTs were visualized *in vitro* in various cell types but they also exist *in vivo* (Gerdes et al., 2013; Ady et al., 2014). In macrophages and HeLa cells, TNT outgrowth can be induced *de novo* by expression of M-Sec (aka TNFaip2), a protein that shows some structural homology with Sec6. M-Sec co-fractionates with Sec6 but does not substitute for Sec6 within the exocyst complex and does not integrate into the exocyst complex. However, M-Sec-induced TNT formation depends on the exocyst complex and on its activator the small GTPase RalA (Hase et al., 2009). The M-Sec-exocyst-Ral association initiates TNT formation, possibly by organizing the actin cytoskeleton but it is not clear at present whether they also participate in TNT fusion with the target cell. M-Sec expression is restricted to myeloid lineages, but the role of the RalA-exocyst axis in TNT induction is likely to be conserved in other cell types (Gousset et al., 2013). One proposed mechanism of TNTs formation is through filopodia conversion (Bukoreshtliev et al., 2009) and the RalA-exocyst interaction is essential for filopodia formation in neurons (Sugihara et al., 2002).

TNTs are also exploited by pathogens to spread and evade antibody neutralization (e.g., HIV, prion). The HIV-1 protein Nef is involved in AIDS pathogenesis notably by promoting nanotube formation. HIV-1 Nef was shown to interact with five members of the exocyst complex and depletion of SEC5 impairs Nef-mediated nanotube formation (Mukerji et al., 2012). Besides a role in TNT formation, the Nef-exocyst interaction regulates the chemotactic response of T lymphocytes to antigen recognition. Surprisingly given the role of both in membrane trafficking, the exocyst is not involved in the perturbation of membrane trafficking by Nef, but in the inhibition of actin remodeling and associated cell spreading (Imle et al., 2015).

### Cancer unravels several aspects of exocyst function and regulation

The canonical role of the exocyst complex in secretion, membrane expansion and delivery of membrane proteins explains in part its involvement in cancer. The invadopodium is an actin-rich, “finger-like,” protrusion of the PM that breaches the extra cellular matrix prior to cancer invasion and metastasis. The exocyst participates in the secretion of degrading matrix metalloproteinases (MMPs) and promotes invadopodium formation by adding new membranes at the tip of the nascent invadopodia. It also controls the Arp2/3-mediated actin polymerization by directly interacting with the Arp2/3 complex and its activator WASH (Liu et al., 2009; Monteiro et al., 2013). In hepatocellular carcinoma cells, the exocyst positions receptors at the PM such as the G-coupled chemokine receptors CXCR4 (Cepeda et al., 2015), whose overexpression is known in a range of tumor types, where it plays a central role in tumor growth and metastasis (Domanska et al., 2013).

The epithelial-mesenchymal transition (EMT) is the process by which epithelial cells lose their interaction with the matrix and neighboring cells and acquire the motile and invasive characteristics of mesenchymal cells. It is involved in cell migration during embryonic development as well as in cancer metastasis. Splice variants of Exo70, generated through the activity of the pre-mRNA splicing factor ESRP1, are involved in EMT (Lu et al., 2013). The level of expression of the mesenchymal isoform of Exo70 correlates with cancer metastasis in the lung of a mouse model and expression of epithelial Exo70 prevents metastasis. Unlike the epithelial isoform, the mesenchymal splice variant of Exo70 interacts with the Arp2/3 complex and stimulates actin branching in lamelipodia and invadopodia, thus promoting motility and invasion. The effect on actin organization, but not secretion, is responsible for this phenotype. Thus, alternative splicing of members of the exocyst complex can change their function dramatically and have implications in disease.

Invasive cancer cells are characterized by an abundance of filopodia, “finger-like” protrusions similar in architecture to invadopodia, that extend from the cell edge and whose functions include cell adhesion and migration. In mice skin melanoma cells, Exo70 induces filopodia formation through its ability to generate negative curvature on the PM in a manner similar to BAR domain proteins (Zhao et al., 2013). This curvature may create a physical space necessary to accommodate the Arp2/3-mediated actin polymerization that drives filopodia outgrowth. On the other hand, over-expression of Exo70 can produce filopodia that are devoid of actin at their most distal tip, suggesting that the membrane deforming activity of Exo70 prevails over its function on actin organization (Zhao et al., 2013). No other exocyst subunit can similarly deform membranes, nor induce filopodia formation. Thus, this membrane-bending activity is probably not a property of the exocyst as a whole, but of a free pool of oligomerized Exo70 (Zhao et al., 2013).

Approximately a third of all human cancers contain oncogenic mutations in the gene encoding the GTPase Ral, which is then produced in its constitutive state (Issaq et al., 2010). Sec5 and Exo84 are effectors of Ral and KD of Sec5 and Exo84 in human embryonic kidney cells (HEK) leads to a reduced Ral-driven tumorigenicity (Issaq et al., 2010). Thus, the role of Ral in tumoriginicity is at least in part through the exocyst complex. In this instance, the Ral-exocyst complex may act as a scaffolding unit that recruits a ikappaB kinase that activates Akt and inhibits apoptosis in cancer cells (Chien and White, 2008). Similarly, KD of Sec8 in flies allows JIP4, a JNK related protein, to interact with proteins in growth signaling pathways, preventing apoptosis (Tanaka et al., 2014). This has important implications in cell immortality which is associated with cancer. The role of the Ral-exocyst axis in abscission may further account for the generation of aneuploidy, a hallmark of cancer (Chen et al., 2006). Finally, in human cell lines, Ral and Exo84 are required for stress-induced autophagy, whose alterations can lead to cancer (Bodemann et al., 2011).

In replicating cells, un-repaired DNA damage is propagated in progeny and commonly leads to cancer. Endogenous and exogenous DNA damage is very frequent and therefore necessitates the existence of a DNA damage response (DDR) machinery that faithfully repairs the DNA. Sec8 has been shown to interact with 33 DDR and chromatin modifying proteins and to modulate the response to DNA damage. Ablation of Sec8 leads to an increase in the frequency of DNA repair but generates chromosomal aberrations (Torres et al., 2015). Therefore, paradoxically, the absence of Sec8 induces genomic instability. Sec8 may participate in the cellular response to DNA damage by restraining the spacio-temporal recruitment of chromatin remodeling proteins in the absence of appropriate DDR signaling, thus promoting a faithful repair. Depletion of Sec8 also confers radioresistance, which has implications for the treatment by radiotherapy of colorectal cancers, in which Sec8 is deleted (Ashktorab et al., 2010; Torres et al., 2015). The G_1_/S checkpoint is another mechanism by which propagation of damaged DNA can be prevented. Sec8 inhibits cell growth and promotes cell-cycle arrest at the G_1_/S phase by controlling p21 expression, a protein that inhibits the activity of cyclins at the G1 checkpoint (Tanaka et al., 2014).

### Immunity and infection

Phagocytosis is a form of endocytosis in which a large, solid particle is recognized, wrapped around an outgrowing host PM and internalized by engulfment. The resulting phagosome maturates into a phagolysosome upon fusion with the acidic lysosomal and endosomal compartments. In mammalian cells, phagocytosis removes invading pathogens and clears apoptotic cells during tissue homeostasis and remodeling. A systematic analysis of the phagosome proteome in drosophila identified members of the exocyst complex (Stuart et al., 2007). In mammalian HeLA cells, depletion of *Exo70* by siRNA is associated with a reduced uptake of large, but not small particles. Whether the whole exocyst is present in nascent phagosomes is controversial and it was suggested that complex assembly and involvement may be transient (Mohammadi and Isberg, 2013).

The exocyst also has a role in phagosome maturation. KD of exocyst proteins in endothelial cells greatly reduces phagosome acidification and bacterial elimination (Figure 3D). Exocyst proteins were observed on Rab11-positive recycling endosomes that dynamically fuse with the phagosome and may mediate the interaction between these two organelles (Rauch et al., 2014) (Figure 3C). These findings further indicate that the exocyst complex associates with and mediates the fusion of intra-cellular compartments and does not solely play a role at the PM.

The exocyst is therefore involved in host defense, but it can on the contrary promote bacteria and parasite entry and invasion. Many Gram-negative intracellular bacterial pathogens use a secretion system to inject virulence factors into the host cell through the formation of a translocation pore. In *Salmonella typhimurium*, one of the members of the T3SS translocation complex, SipC, directly interacts with actin and with several members of the exocyst complex. The recruitment of the exocyst at the invadosomes, the sites of invasion, is thought to increase membrane expansion and create a membrane ruffling that will lead to the internalization of *Salmonella* by macropinocytosis. In support of this, KD of *Sec5* reduces membrane ruffling and bacterial invasion (Nichols and Casanova, 2010).

*Trypanosoma cruzi* is an obligate intracellular parasite responsible for Chagas disease that causes damage to the heart and central nervous system. During invasion, the parasite makes a parasitophorous vacuole (PV) in which it will reside and develop, protected from the host's lysosome. In the process, *T. cruzi* injures the host's PM, opening large wounds through which extra cellular calcium can engulf. This sudden increase in calcium influx triggers a burst in calcium-mediated exocytosis of lysosomes, which, upon fusion with the PM and active removal of lesions by endocytosis, participate in wound healing and PM repair (Andrews et al., 2014). Conversely, PM repair is required for *T. cruzi* invasion. The PV is in fact derived from the host's endosomal or lysosomal compartment (Fernandes et al., 2011). Exo70 and Sec8 were shown to accumulate in nascent *T. cruzi* vacuoles and at the sites of wounding and to be required for both PM repair and *T. cruzi* invasion (Fernandes et al., 2015). This study raises the possibility that the exocyst, which is traditionally associated with constitutive exocytosis, is also involved in Ca^2+^-mediated, regulated exocytosis. This would have implications for wound repair in other tissues. In budding yeast, damage of the cell wall and PM triggers the reorientation of the actin cytoskeleton and exocyst complex from sites of polarized growth to the wounded site, thus avoiding competition between cell polarization and wound healing (Kono et al., 2012).

Pathogens use the recycling pathway and the exocyst complex to exit infected cells and spread to neighboring cells. *Porphyromonas gingivalis* is a bacterium pathogen that penetrates gingival epithelial cells by exploiting the endocytic pathway of its host. Expression of dominant negative versions and KD of exocyst components compromise the recycling of the pathogen back to the PM, its exit from the host, and therefore its spreading to neighboring cells (Takeuchi et al., 2011).

Interestingly, the genomes of a few parasitic protists and ciliates contain no or a reduced number of exocyst subunits (Koumandou et al., 2007; Klinger et al., 2013), indicating that the exocyst is not absolutely required for cellular life. However, it was suggested that this is not a consequence of evolution but an adaptation to the lifestyle and anatomy of these parasites in which directed exocytosis can occur without any specialized machinery (Klinger et al., 2013).

## Conclusion

Membrane expansion, cell polarity and cell division are key fundamental cellular processed that are central in all kingdoms for tissue development and cell function. It is therefore not surprising that the mechanisms and factors that orchestrate these processes have been conserved across evolution. Besides, the mechanisms of pathogen infection or cell invasion are rather similar between kingdoms. The fact that the exocyst complex is essential for survival in fungi and animals, along with the fact it is linked to a number of diseases, confirms its pivotal and universal role.

Although very few complementation experiments have been carried out, the composition and function of individual sub-units would seem to be essentially conserved from plants to animals, despite little primary sequence similarities. Multi-cellular organisms have developed different ways to deal with tissue specificity and needs for differential regulation. Splice variants probably account for this specialization in plants and metazoan, but plants also have paralogs that are expressed from distinct loci (Cvrckova et al., 2012; Lu et al., 2013). Either way, the implications of this diversification are currently far from being clear.

The exocyst is made of eight proteins and the full octameric complex clearly assembles in all species. Whether sub-complexes exist is a matter of debate and may be species-dependent. The idea that sub-complexes may exist originally came from studies of *S. cerevisiae* (Boyd et al., 2004). However, no stable sub-complex has so far been purified biochemically from this yeast, but a stable holocomplex was (Heider et al., 2016). In filamentous fungi, plants and animals, the dilemma comes from observations that different exocyst members localize to different sub-cellular locations. In higher eukaryotes few studies have systematically reported the localization of all subunits in the same cell, under the same experimental conditions. Evidence that some exocyst members at least are localized to distinct cellular regions is however convincing. The existence of combinations of exocyst components could be a mean to provide functional diversity in these complex systems. This later hypothesis would explain why the individually KO of all sub-units in plants and animals does not give rise to the same exact phenotype.

Research in each kingdom has focussed on different aspects of exocyst biology but similarities and differences clearly exist that could be exploited for a better understanding of this complex in health and disease.

## Author contributions

MMU wrote the chapter on fungi. MD wrote the chapter on plants. CH helped write the chapter on animals. HD helped write the chapter on cilia. IJ wrote most of the chapter on animals, made the figures and coordinated the preparation of the ms.

### Conflict of interest statement

The authors declare that the research was conducted in the absence of any commercial or financial relationships that could be construed as a potential conflict of interest.
